# Impaired Mitochondrial Translation and Bioenergetic Dysfunction Associated with the *MT-RNR1* m.1014A>G Variant

**DOI:** 10.3390/ijms27219513

**Published:** 2026-10-24

**Authors:** Irene Marrone, Blendi Ura, Fulvio Celsi, Luisa Zupin, Eduardo Maria Sommella, Valentina Golino, Paolo Peruzzo, Chiara Ciardi, Maria Teresa Bonati, Elena Genova, Massimo Zeviani, Alessandro Mauro Spinelli, Valeria Capaci

**Affiliations:** 1 Institute for Maternal and Child Health—IRCCS “Burlo Garofolo”, Via dell’Istria 65, 34137 Trieste, Italyfulvio.celsi@burlo.trieste.it (F.C.); luisa.zupin@burlo.trieste.it (L.Z.); elena.genova@burlo.trieste.it (E.G.);; 2 Department of Pharmacy, University of Salerno, 84084 Fisciano, Italy; 3 National PhD Program in RNA Therapeutics and Gene Therapy, University of Naples Federico II, 80138 Napoli, Italy; 4 Regional Coordinating Center for Rare Diseases, 33100 Udine, Italy; 5 Neuroradiology Unit, University Hospital, 33100 Udine, Italy; chiara.ciardi@asufc.sanita.fvg.it

**Keywords:** *MT-RNR1*, mitochondrial disease, mitoribosome, translation defect, bioenergetics

## Abstract

Pathogenic variants in *MT-RNR1* are classically associated with aminoglycoside-induced sensorineural hearing loss through disruption of the mitochondrial 12S rRNA decoding center. Their contribution to pediatric neurological disease remains poorly defined. We identified a novel homoplasmic *MT-RNR1* variant, m.1014A>G, in a patient with drug-resistant focal epilepsy beginning at 9 months of age who remained undiagnosed after trio whole-exome sequencing filtered using an extensive epilepsy gene panel. The variant was also detected in her mother. Structural modeling suggests that the variant, affecting a highly conserved region of the mitochondrial 12S rRNA, may alter the mitochondrial small ribosomal subunit assembly. To investigate its pathogenicity, we performed comprehensive functional studies in patient-derived fibroblasts. Unlike canonical *MT-RNR1* variants, m.1014A>G did not confer aminoglycoside toxicity. Functional in vitro studies on patients’ fibroblasts demonstrated increased 12S/16S rRNA ratios, reduced abundance of both mitoribosomal subunit proteins, decreased levels of mitochondrially encoded respiratory-chain subunits, and impaired oxidative phosphorylation. These abnormalities were accompanied by mitochondrial network fragmentation, increased mitochondria–lysosome colocalization suggestive of enhanced mitophagy and reduced mitochondrial DNA copy number. Altogether, the findings are consistent with defective mitoribosome assembly and altered mitochondrial translation, resulting in a bioenergetic defect. Our study points to a previously unrecognized candidate pathogenic mechanism associated with *MT-RNR1* and may expand the phenotypic spectrum of *MT-RNR1*-related disorders to include maternally inherited infantile-onset epileptic encephalopathy. These findings highlight the importance of dedicated mitochondrial DNA analysis in children with unexplained early-onset epilepsy despite negative nuclear genomic testing.

## 1. Introduction

Epilepsy affects approximately 50 million individuals worldwide, and nearly one-third of patients fail to achieve adequate seizure control despite appropriate pharmacological treatment, developing drug-resistant epilepsy [1,2]. In the pediatric population, early-onset epileptic and developmental encephalopathies represent a particularly severe clinical condition. Seizure onset during infancy or early childhood not only reflects underlying severe neurological dysfunction, but also interferes with critical stages of brain development, resulting in progressive cognitive, motor, and behavioral impairment [3,4]. In this subgroup of patients, identification of the underlying genetic etiology is crucial to guide therapeutic strategies, avoid potentially harmful treatments, provide prognostic information, and enable accurate genetic counseling and recurrence risk assessment for affected families [5]. Nevertheless, early-onset epileptic and developmental encephalopathies remain diagnostically challenging disorders, as the causative genetic defect frequently remains undetected despite extensive investigations [6].

One of the possible underlying causes of epilepsy may be related to mitochondrial defects. Indeed, neurological manifestations, including epilepsy, developmental regression, and encephalopathy, are particularly prominent in children with mitochondriopathies due to the high energy demands of the developing brain [7,8].

Mitochondrial diseases constitute a genetically and clinically heterogeneous group of disorders caused by pathogenic variants affecting either the mitochondrial genome (mtDNA) or nuclear genes involved in mitochondrial function, ultimately impairing oxidative phosphorylation (OXPHOS) and cellular energy homeostasis. With an estimated prevalence of approximately 1 in 5000 individuals, mitochondrial disorders are among the most common inherited metabolic diseases and represent a major cause of pediatric neurodegenerative disease [9,10].

The nervous system is especially vulnerable to mitochondrial dysfunction, impairing OXPHOS and altered cellular energy homeostasis, due to its substantial metabolic requirements [11]. Epilepsy is a well-recognized feature of several mitochondrial syndromes, including Mitochondrial Encephalomyopathy, Lactic Acidosis, and Stroke-like episodes (MELAS), Myoclonic Epilepsy with Ragged Red Fibers (MERRF) and Leigh syndrome, and may occasionally represent the predominant or even isolated neurological manifestation associated with pathogenic nuclear and mtDNA variants [11]. A distinctive characteristic of mitochondrial genetics is the marked phenotypic variability observed among affected individuals, influenced by factors such as heteroplasmy levels, tissue-specific segregation of mtDNA, and nuclear genetic background [12]. However, even homoplasmic mtDNA variants may lead to highly variable clinical phenotypes within the same family, as demonstrated in maternally inherited aminoglycoside-induced deafness associated with *MT-RNR1* variants [12,13].

The mitochondrial genome (mtDNA) encodes 13 essential protein subunits of the oxidative phosphorylation (OXPHOS) system, all of which are exclusively translated by the mitochondrial ribosome (mitoribosome). The mitoribosome consists of two subunits, the small subunit (mt-SSU) and the large subunit (mt-LSU), which together mediate the synthesis of mtDNA-encoded OXPHOS proteins. Among mtDNA genes, *MT-RNR1* and *MT-RNR2* encode the 12S and 16S ribosomal RNAs, respectively, which are essential structural and functional components of the mitoribosome. Proper ribosomal folding and assembly are crucial for mitochondrial translation and normal respiratory chain function. Accordingly, defects affecting mitoribosomal components, including mitochondrial ribosomal RNA and proteins, are associated with mitochondrial disorders such as Perrault syndrome and other neurodevelopmental diseases [14].

Established pathogenic *MT-RNR1* variants, most notably m.1555A>G, but also m.1494C>T and m.1095T>C, are strongly associated with non-syndromic sensorineural hearing loss and severe aminoglycoside-induced ototoxicity. These variants alter the decoding center of the 12S rRNA, increasing its structural similarity to bacterial ribosomal RNA and thereby enhancing aminoglycoside binding, which impairs translational fidelity and exacerbates mitochondrial dysfunction [15,16]. Beyond aminoglycoside hypersensitivity, emerging evidence suggests that *MT-RNR1* variants may contribute to disease through additional pathogenic mechanisms, including altered rRNA secondary structure, defective mitoribosomal assembly, and impaired mitochondrial translation. These abnormalities ultimately compromise the synthesis of OXPHOS subunits and interfere with respiratory chain function, leading to mitochondrial dysfunction and tissue-specific clinical phenotypes [17].

Additional mitochondrial DNA analysis is therefore essential in the diagnostic workup of patients with unexplained epilepsy and neurodevelopmental disorders, particularly when nuclear genetic testing is unrevealing [8].

Here, we report a patient with severe early-onset drug-resistant epilepsy who remained without a definitive molecular diagnosis for over three decades. Dedicated mtDNA analysis identified a novel homoplasmic *MT-RNR1* variant, m.1014A>G, also present in her mother. Functional studies in patient-derived fibroblasts revealed a mitoribosomal defect, supporting a possible pathogenic role of the variant. This case underscores the importance of mtDNA analysis in developmental and epileptic encephalopathies and expands the phenotypic spectrum associated with *MT-RNR1* variants beyond aminoglycoside-related hearing loss.

## 2. Results

### 2.1. Clinical Presentation and Diagnostic Evaluation

The index case (IV-1; hereafter named MT24) is a 41-year-old woman affected by infantile-onset, drug-resistant focal epilepsy which currently meets the ILAE diagnostic criteria for Lennox–Gastaut syndrome.

She was born at 36 weeks of gestation following placental abruption, without documented neonatal distress. Seizure onset occurred at 9 months of age and was initially characterized by generalized tonic–clonic seizures. At 14 years of age, the patient underwent right fronto-insular lesionectomy and corticectomy for lesions interpreted as severe cortical dysplasia with balloon cells. Surgery resulted in a transient reduction in seizure frequency; however, epilepsy eventually progressed.

At that time, based purely on histopathology of the surgical specimens, a diagnosis of tuberous sclerosis had been proposed. However, our re-evaluation some 25 years later has ruled out that current international diagnostic criteria for tuberous sclerosis complex (TSC) are met. Also, the more infrequent scenario of mosaicism confined to the CNS seemed not convincing at all, as no further area of FCD has been found in high-field neuroimaging.

The clinical pattern of seizures has now evolved into daily seizures of multiple types, including drop attacks and focal tonic seizures with impaired awareness. A moderate left spastic hemiparesis is the late consequence of surgery.

Electroencephalogram recordings taken over time demonstrated a globally slowed and asymmetric background with multifocal epileptiform discharges predominantly involving bilateral fronto-temporal regions.

The most recent brain MRI revealed extensive right hemispheric structural abnormalities, including localized post-surgical encephalomalacia, gliosis of the fronto-parieto-temporal and capsulo-nuclear regions, secondary ventricular enlargement, size reduction of the right thalamus, thinning of the ipsilateral cerebral peduncle, and diffuse hyperintensity on Fluid-Attenuated Inversion Recovery (FLAIR) images along the corticospinal tract in the right bulbo-pontine region, suggesting Wallerian degeneration. Spectroscopy and non-contrast Magnetic Resonance Angiography sequences showed no abnormalities.

Transthoracic echocardiogram, standard electrocardiogram, pure-tone audiometry, complete ophthalmological examination, electromyography and nerve conduction studies of the four limbs, laboratory assessment of ovarian, liver, and kidney function, bone densitometry, dermatological examination, and fasting plasma amino acid profile did not lead to the discovery of any abnormalities suggestive of mitochondrial disease.

The mother of the index case presented a personal history of intact pontomesencephalic cavernous angioma, a previous hemorrhagic cerebellar stroke at the age of 20 (presumably caused by an additional cavernoma and now resulting in right-sided cerebellar porencephaly and ipsilateral spastic ataxia which, however, has been very slowly progressive at least over the course of the last two decades, contrary to expectations), anxiety/depression, sleep disturbances, and chronic macrocytosis without anemia. The connection between these clinical data, the described variant, and family history is to be regarded as uncertain, to the best of our knowledge.

### 2.2. Identification of a Rare MT-RNR1 Variant

Genetic testing was requested because of the presence of a neurodevelopmental disorder associated with epilepsy. Trio whole-exome sequencing (WES), with variant filtering based on an approximately 1688-gene panel associated with neurodevelopmental disorders and epilepsy, did not identify pathogenic or likely pathogenic variants in nuclear genes according to American College of Medical Genetics and Genomics (ACMG) criteria [18]. No clinically significant copy number variants were detected.

However, mitochondrial DNA analysis identified in the proband the homoplasmic m.1014A>G variant in the *MT-RNR1* gene, encoding mitochondrial 12S rRNA (576/576 sequencing reads). Subsequent analysis demonstrated that the mother was also homoplasmic for the same variant in blood-derived DNA (III-2, hereafter MT24M; 422/422 sequencing reads). No DNA samples from other maternal relatives were available for segregation analysis (the maternal grandmother, II-2, and the maternal aunt, III-3, who died at 8 years of age of unknown causes); therefore, it was not possible to establish whether the variant was transmitted through the maternal lineage or arose de novo in the mother’s germline.

The m.1014A>G variant is not reported in the Clinvar (https://www.ncbi.nlm.nih.gov/clinvar/, accessed on 1 September 2026), gnomAD-mtDNA (https://gnomad.broadinstitute.org/, accessed on 1 September 2026) and HelixMTdb databases (https://www.helix.com/mitochondrial-variant-database, accessed on 1 September 2026). In MITOMAP, the variant is reported at an extremely low frequency (0.003%), with a conservation score of 97.78%, and no pathogenicity predictions are currently available from MitoTIP, HmtVar, or APOGEE2 (https://www.mitomap.org/MITOMAP, accessed on 1 September 2026). To date, the variant has only been described in a population genetics study investigating haplotypes in indigenous Siberian populations [19]. Collectively, these findings support the rarity of the variant and prompted further functional investigations to assess its potential pathogenicity.

### 2.3. The m.1014A>G Variant Does Not Confer Hypersensitivity to GENTAMICIN

Pathogenic *MT-RNR1* variants, in particular m.1555A>G, m.1494C>T, and m.1095T>C, are frequently associated with aminoglycoside hypersensitivity [20,21,22]. We therefore investigated whether the m.1014A>G variant could alter cellular sensitivity to gentamicin.

**Figure 1:**
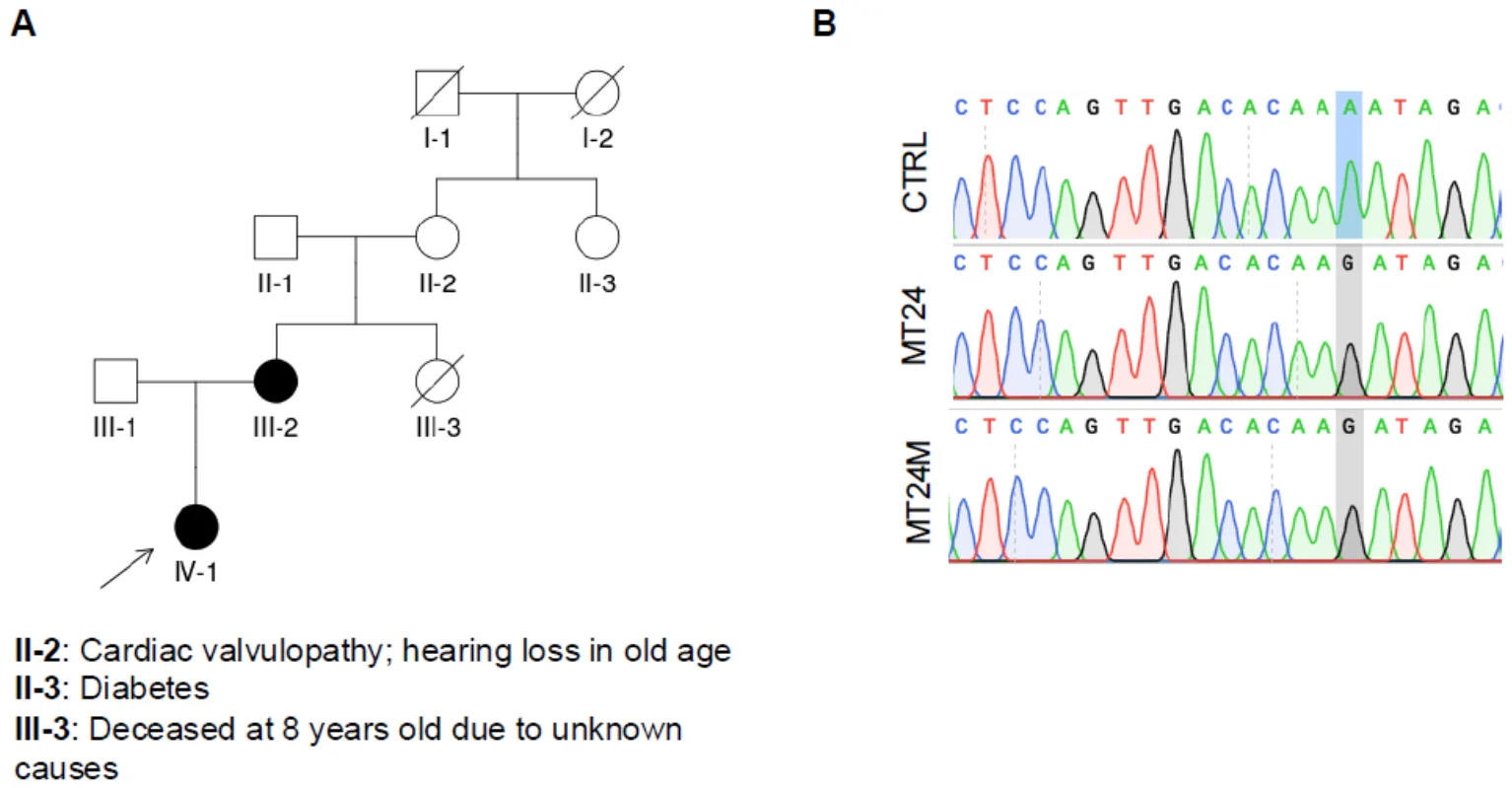
Identification and genetic context of the m.1014A>G variant. (**A**) Pedigree of the family. The filled black symbol (IV-1) indicates the proband carrying the homoplasmic m.1014A>G variant in the *MT-RNR1* gene. The arrow denotes the proband. The pedigree was drawn using QuickPed version 4.5.0 [23]. (**B**) Sanger sequencing chromatograms of the mitochondrial position m.1014 in DNA from primary fibroblasts of the control (CTRL), proband (MT24), and her mother (MT24M). The blue highlight marks the reference adenine (A) in the control and the homoplasmic guanine (G) substitution in MT24 and MT24M.

**Figure 2:**
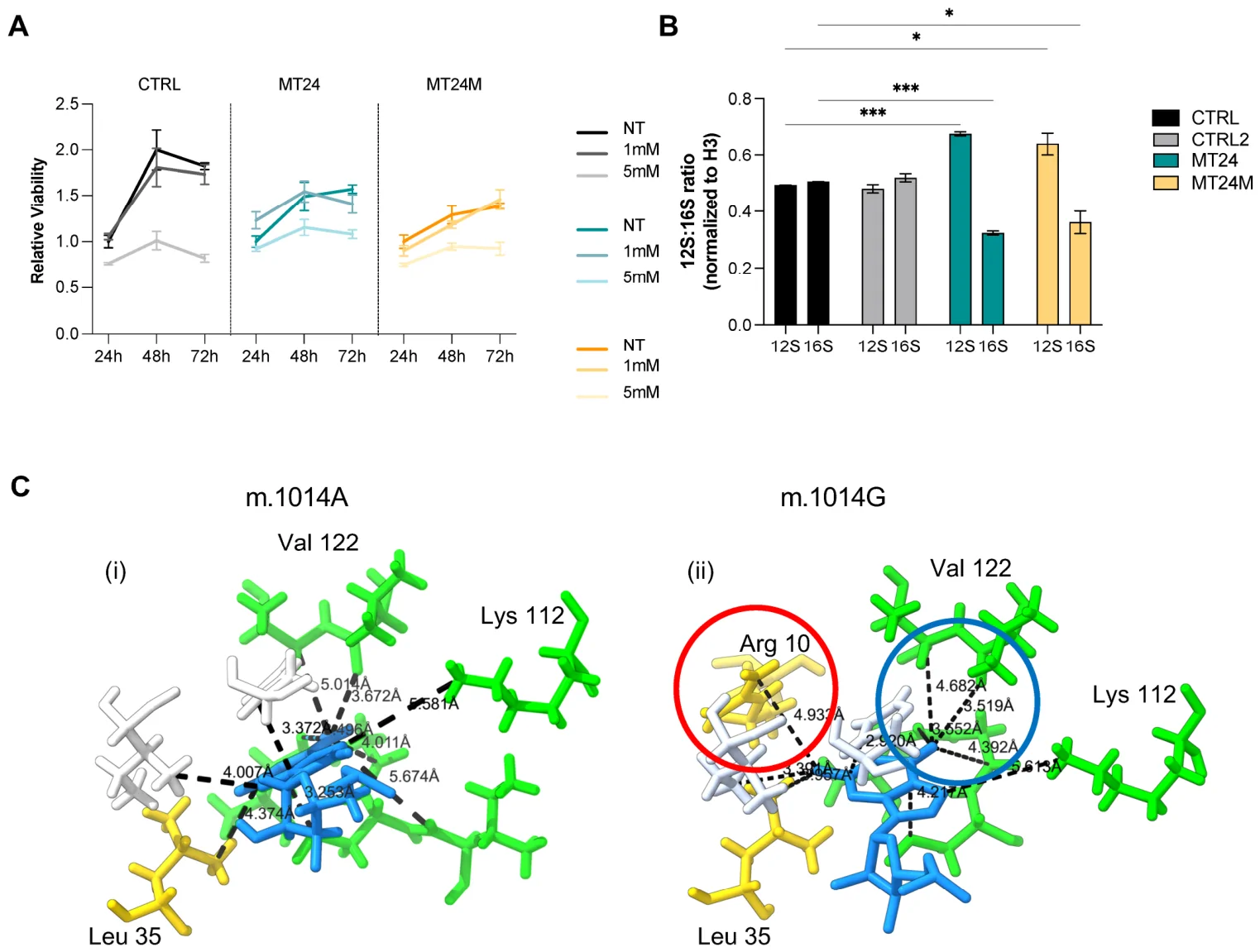
Effect of the m.1014A>G variant on gentamicin sensitivity and 12S mt-rRNA levels. (**A**) Cell viability assessed by Crystal Violet staining of primary human dermal fibroblasts (HDFs) derived from controls (CTRL), the proband (MT24), and the mother (MT24M) treated with gentamicin at 0 (NT), 1 mM, and 5 mM for 24, 48, and 72 h. Relative viability is normalized to untreated cells at each time point. Data represent mean ± SEM of three independent experiments. (**B**) Expression levels of 12S and 16S mt-rRNA determined by quantitative RT-PCR in primary HDFs from CTRL, CTRL2, MT24, and MT24M, normalized to histone H3. The 12S:16S ratio is shown for each cell line. Data represent mean ± SEM. Asterisks indicate statistical significance (*** *p* < 0.001; * *p* < 0.05; unpaired Welch’s *t*-test). (**C**) Structural modeling of the m.1014A>G variant in the human 12S mt-rRNA secondary structure, and 3D reconstruction of neighborhood (max 6 Å distance) amino acids surrounding position 1014 of the 12S rRNA sequence, based on 7QI4 structures from RCS pdb. In blue is the nucleotide in position 1014 of chain AA (12s rRNA), in (i) wild type (A), and mutated (G), in (ii). Green, amino acids pertaining to chain AP; white, those pertaining to chain AQ; and gold, those pertaining to chain AI (chain codes according to 7QI4 structure). Distances between the nucleotide and the amino acids are indicated as dashed lines. The red circle in (ii) shows a “new” amino acid nearer compared to wild-type structures, while the blue circle in B displays an example of shortened distances.

Primary fibroblasts were obtained from skin biopsies of the proband (MT24) and her mother (MT24M). Sanger sequencing of fibroblast-derived DNA confirmed that both cell lines harbored the m.1014A>G variant in the homoplasmic state (Figure 1B). Cell viability was assessed following 24, 48 and 72 h exposure to increasing concentrations of gentamicin (1 and 5 mM). Although prolonged gentamicin treatment induced a time-dependent reduction in viability in all fibroblast lines, and mutant cells showed reduced viability, no significant differences were observed between mutant and control cells upon gentamicin treatment (Figure 2A; raw absorbance values are reported in Table S1). These findings indicate that, unlike canonical aminoglycoside-sensitive *MT-RNR1* variants, m.1014A>G does not confer increased gentamicin susceptibility.

### 2.4. The m.1014A>G Alters the 12S mt-rRNA Levels

To investigate the structural basis of this defect, we generated a static in silico substitution model of the m.1014A>G variant using the human mitoribosome cryo-EM structure (PDB 7QI4) as a wild-type reference, visualized in UCSF ChimeraX [24,25]. Comparison of the local environment (6 Å) surrounding nucleotide 1014, located within helix H23 of the 12S rRNA central domain, revealed a more compact and less accessible interface in the mutant model, including a newly formed close contact with the N-terminal region of the mt-SSU platform protein bS21m (MRPS21) (Figure 2C).

Given the critical role of 12S rRNA in assembly and function of the mitochondrial small ribosomal subunit, we investigated whether the variant affected mitochondrial rRNA levels. We quantified 12S and 16S rRNA levels, encoded by *MT-RNR1* and *MT-RNR2* respectively, in primary fibroblasts from MT24 and MT24M compared to healthy controls. The 12S:16S rRNA ratio was calculated using relative expression values normalized to H3 expression [26,27]. Interestingly, patient-derived fibroblasts exhibited a significant increase in the 12S:16S rRNA ratio compared with controls (Figure 2B). To exclude the possibility that this increase resulted from accumulation of degradation intermediates, two independent primer pairs targeting the 5′ and 3′ regions of the transcript were used, yielding consistent results (Supplementary Figure S1).

These findings suggest altered stability, turnover, or assembly dynamics of the mitochondrial small ribosomal subunit associated with the m.1014A>G variant.

### 2.5. Mito-Proteomic Analysis Reveals a Defect in Mitoribosome Homeostasis and Mitochondrial Translation

As suggested by structural modeling (Figure 2C), this region of the 12S rRNA is in close proximity to platform-associated mt-SSU proteins; we next investigated whether the m.1014A>G variant affected mitoribosome composition and mitochondrial proteostasis. Mitochondria were isolated from primary fibroblasts and analyzed by quantitative mass spectrometry-based proteomics. Global proteomic analysis identified and quantified 1140 proteins, corresponding to the whole mitochondrial proteome [28,29]. Functional enrichment analysis using Gene Ontology and Reactome pathway annotation confirmed significant representation of mitochondrial pathways and respiratory chain components (Supplementary Figure S2A; Table S2).

**Figure 3:**
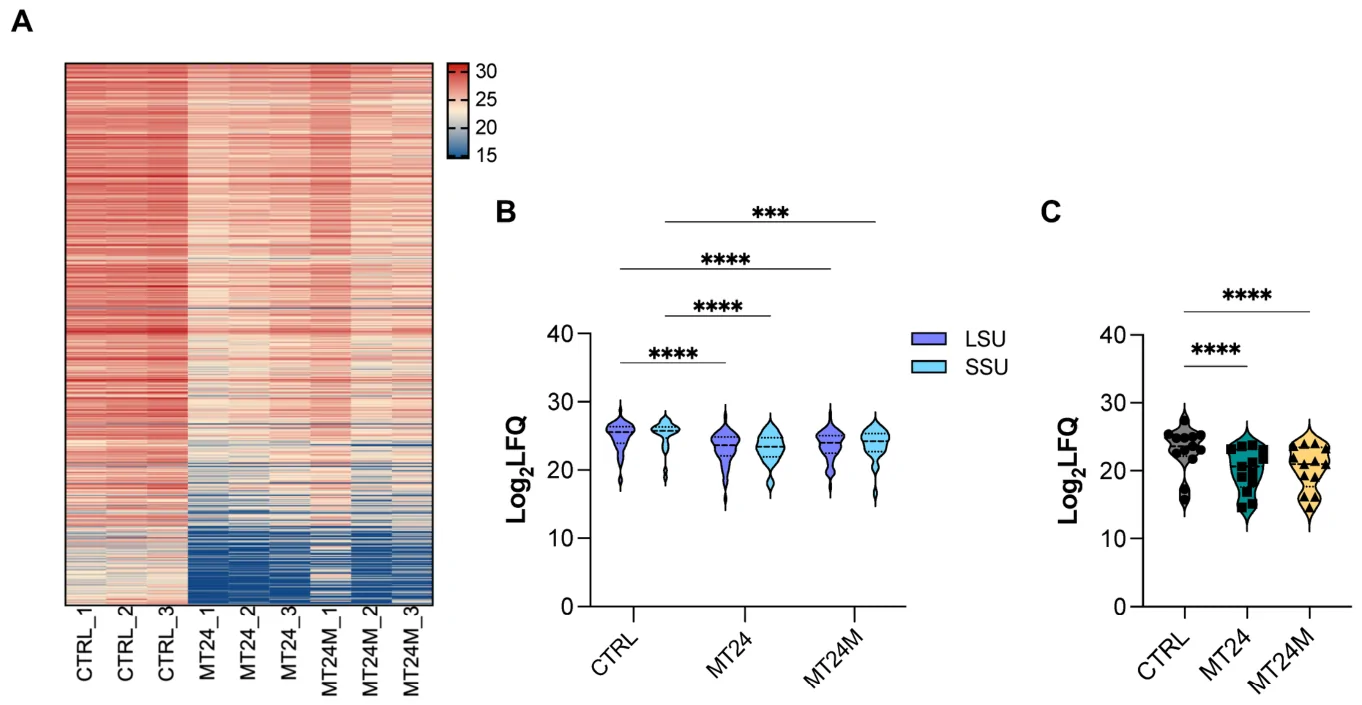

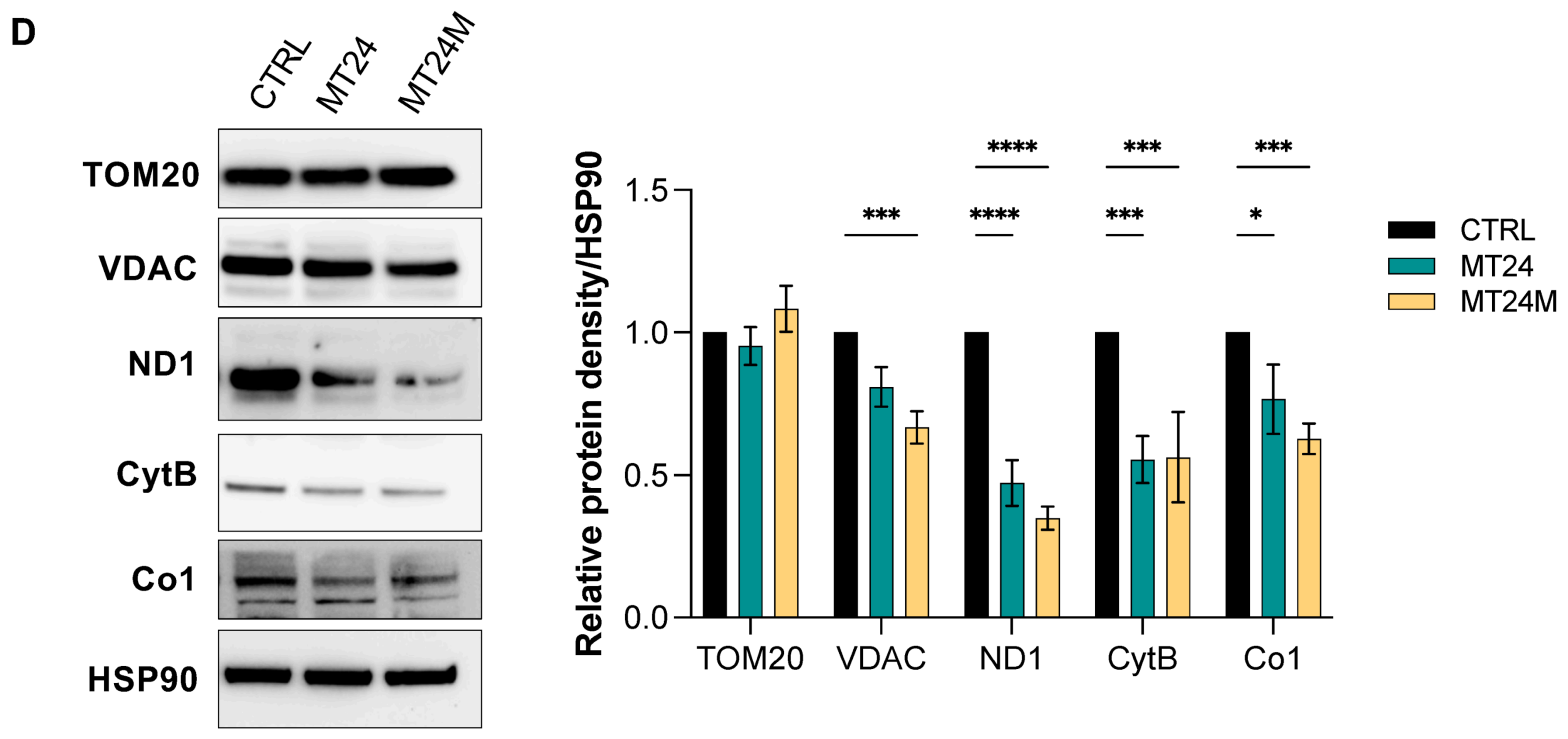
Mitoproteomic analysis and mitochondrial protein expression. (**A**) Heatmap of quantitative proteomic data showing Log_2_ LFQ (label-free quantification) intensity values across all biological replicates (CTRL_1–3, MT24_1–3, MT24M_1–3). Rows represent individual proteins; columns represent biological replicates. Color scale ranges from blue (low intensity) to red (high intensity). (**B**) Violin plots of Log_2_ LFQ intensities of mitoribosomal large subunit (LSU, violet) and small subunit (SSU, light blue) proteins in CTRL, MT24, and MT24M. (**C**) Violin plots of Log_2_ LFQ intensities of 13 mtDNA-encoded OXPHOS subunits in CTRL, MT24, and MT24M. (**D**) Representative Western blot analysis of mitochondrial proteins (TOM20, VDAC, ND1, CytB, Co1) in primary HDFs from CTRL, MT24, and MT24M. HSP90 was used as the loading control. Bar graphs show relative protein density (mean ± SEM, *n* = 3 independent experiments), normalized to HSP90 and expressed relative to CTRL. Statistical significance was assessed by 2-way ANOVA and multiple comparison with Dunnett’s correction. (**** *p* < 0.0001; *** *p* < 0.001; * *p* < 0.05).

Differential expression analysis revealed widespread downregulation of mitochondrial proteins in mutant fibroblasts. Specifically, 492 proteins were significantly downregulated in MT24 cells, whereas 368 proteins were downregulated, and only one was upregulated in MT24M cells (Figure 3A; Table S2).

Remarkably, nearly all detected proteins constituting the mitochondrial small ribosomal subunit (mt-SSU) showed reduced abundance in both mutant fibroblast lines. A concomitant reduction in proteins belonging to the large mitochondrial ribosomal subunit (mt-LSU) was also observed, although the effect was more pronounced for the small subunit. Overall quantification of mitoribosomal proteins demonstrated that MT24 and MT24M displayed the lowest mt-SSU and mt-LSU levels compared to control cells, indicating a defect in mitoribosome assembly in both the proband and the mother, although the impairment appeared milder in the latter (Figure 3B, Table S3).

In parallel, proteomic analysis revealed a marked reduction in mitochondrial encoded respiratory complex subunits (Figure 3C; Supplementary Figure S2B), consistent with impaired mitochondrial translation.

To validate the proteomic findings, we analyzed representative mitochondrially encoded respiratory chain proteins by Western blot. Specifically, we assessed NADH Dehydrogenase Subunit 1 (ND1), Cytochrome b (CYTB), and Cytochrome c Oxidase Subunit I (COX1), which are representative subunits of Complexes I, III, and IV, respectively, together with the nuclear-encoded mitochondrial proteins Translocase of Outer Mitochondrial Membrane 20 (TOM20) and Voltage-Dependent Anion Channel (VDAC). Western blot analysis confirmed a significant reduction in mitochondrially encoded proteins in fibroblasts carrying the m.1014A>G variant, whereas TOM20 levels remained largely preserved. VDAC showed a mild reduction, suggesting additional alterations in mitochondrial homeostasis (Figure 3D).

Collectively, these data support the presence of impaired mitoribosome integrity and defective mitochondrial translation associated with the m.1014A>G variant.

### 2.6. The m.1014A>G Variant Affects Mitochondrial Respiration

**Figure 4:**
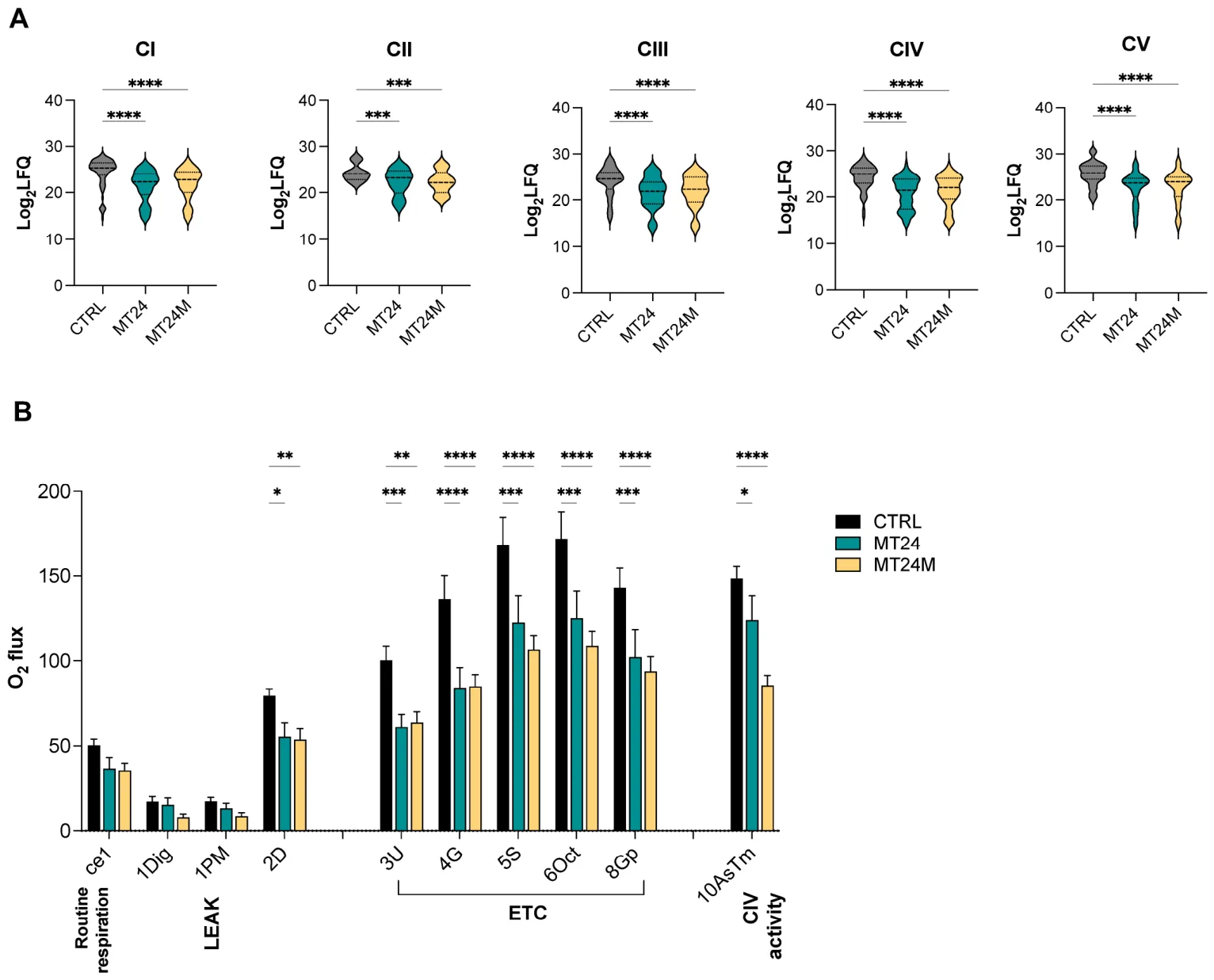
OXPHOS complex proteomics and mitochondrial respirometry. (**A**) Violin plots of Log_2_ LFQ intensities of proteins assigned to respiratory chain Complexes I (CI), II (CII), III (CIII), IV (CIV), and V (CV) in CTRL, MT24, and MT24M. (**B**) High-resolution respirometry showing specific O_2_ flux in digitonin-permeabilized primary HDFs from CTRL, MT24, and MT24M, measured by the substrate–uncoupler–inhibitor titration (SUIT) protocol. Steps shown: routine respiration (ce1), LEAK state (1Dig, 1PM), electron transport chain (ETC) capacity (3U, 4G, 5S, 6Oct, 8Gp), and Complex IV activity (10AsTm). Data represent mean ± SEM of three independent experiments. Statistical significance was assessed by 2-way ANOVA and multiple comparison with Dunnett’s correction (**** *p* < 0.0001, *** *p* < 0.001, ** *p* < 0.01, * *p* < 0.05).

Interestingly, proteomic analysis revealed a global reduction respiratory chain complex subunit (Figure 4A, Tables S4–S8). We next investigated the impact of the m.1014A>G variant on mitochondrial respiration in human dermal fibroblasts (HDFs) using high-resolution respirometry performed with the Oroboros O2k oxygraph. This analysis revealed a significant reduction in oxygen consumption in 12S rRNA–mutant primary HDFs compared with wild-type control cells.

Specifically, routine respiration and non-phosphorylating resting respiration (LEAK respiration, measured after the addition of pyruvate and malate) did not differ significantly between the two groups. In contrast, OXPHOS capacity, assessed through NADH-linked respiration at Complex I following the addition of ADP, was significantly reduced in both MT24 and MT24M fibroblasts. Similarly, maximal electron transfer system capacity, evaluated under uncoupled conditions, was markedly decreased relative to controls. This reduction persisted across all subsequent respiratory states, indicating a generalized impairment across downstream respiratory complexes (including Complex II-linked respiration and Complex IV activity) (Figure 4B).

These findings indicate that fibroblasts carrying the m.1014A>G variant display impaired oxidative phosphorylation capacity and mitochondrial electron transport chain function.

### 2.7. The m.1014A>G Variant Is Associated with Reduced Mitochondrial Mass, Altered Mitochondrial Network Organization and Increased Mitochondria–Lysosome Colocalization

So far, proteomic analysis revealed a widespread reduction not only in mitoribosomal and OXPHOS subunits, but also in several structural mitochondrial proteins, including the outer membrane marker VDAC, suggesting a global reduction in mitochondrial mass, possibly secondary to the translational defect. To investigate this possibility, we assessed mitochondrial network morphology and mitochondria–lysosome association as an indirect readout of mitophagy.

**Figure 5:**
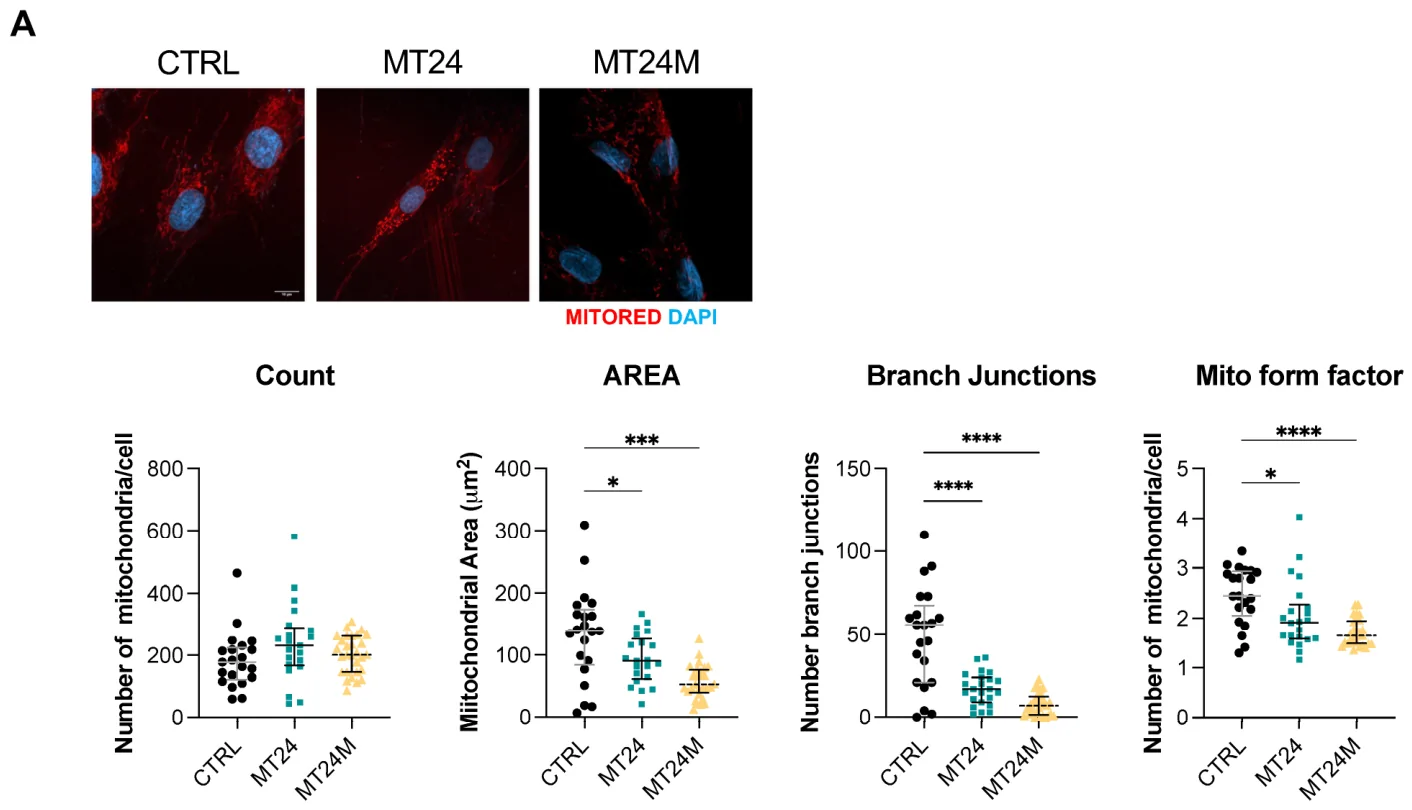

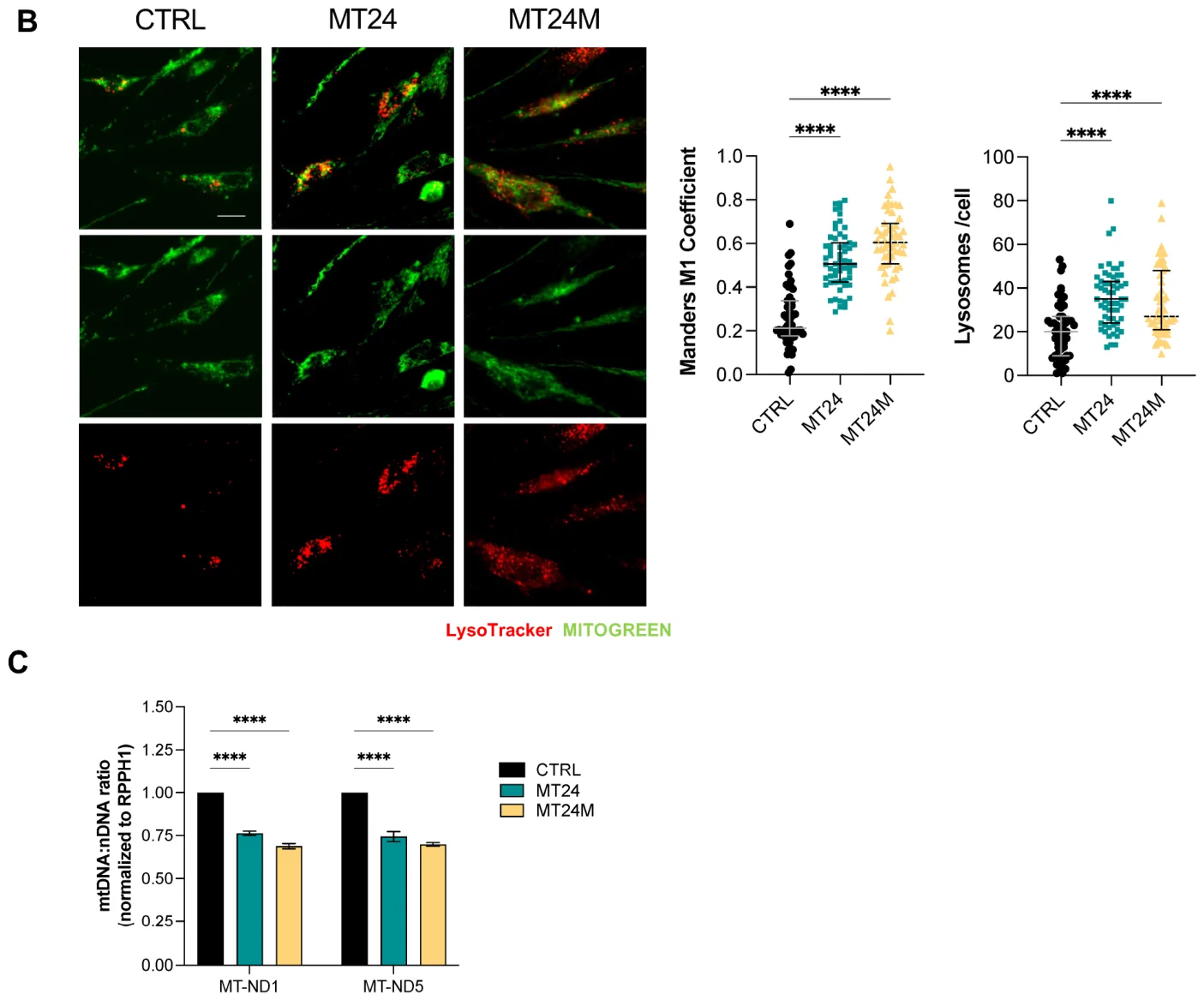
Mitochondrial network morphology, mitophagy, and mtDNA content. (**A**) Representative confocal microscopy images of primary HDFs from CTRL, MT24, and MT24M stained with MitoTracker Red (MITORED, red) and counterstained with DAPI (blue) to visualize nuclei. Scale bar, 10 µm. Scatter plots show quantification of mitochondrial count, area (µm^2^), branch junctions, and mitochondrial form factor per cell, calculated using the Mitochondrial Analyzer plugin for ImageJ. *n* = 50 cells per condition. (**B**) Representative fluorescence microscopy images of primary HDFs from CTRL, MT24, and MT24M stained with MitoTracker Green (green) and Lysotracker (red). Scatter plots show quantification of mitochondria–lysosome colocalization, expressed as Manders’ coefficient M1 (fraction of LysoTracker signal overlapping MitoTracker Green signal) calculated using the JACoP plugin for Fiji. Scale bar, 20 µm. *n* = 50 cells per condition. (**C**) Mitochondrial DNA (mtDNA) copy number assessed by quantitative PCR using primers targeting mitochondrially encoded genes *MT-ND1* and *MT-ND5*, normalized to the nuclear reference gene *RPPH1*. The mtDNA:nDNA ratio is shown for CTRL, MT24, and MT24M. Data represent mean ± SEM, *n* = 3 independent experiments. Statistical significance: * *p* < 0.05, *** *p* < 0.001, **** *p* < 0.0001 (2-way ANOVA).

Accordingly, mitochondrial staining with Mitotracker and confocal microscopy analysis demonstrated marked changes in mitochondrial morphology in patient-derived fibroblasts. Mitochondria appeared more punctate, with shorter tubules and reduced branching. Notably, no significant differences were observed in mitochondrial number. In contrast, the mitochondrial area and form factor (parameters reflecting mitochondrial size and network complexity) were significantly reduced in patient cells, indicating smaller and more fragmented mitochondria. Consistently, network analysis revealed a reduction in both branch number and branch junctions in mutant cells (Figure 5A). Together, these findings indicate that the m.1014A>G variant is associated with marked alterations in mitochondrial morphology and network connectivity.

In addition, Mitotracker and LysoTracker co-staining revealed a marked increase in mitochondria–lysosome colocalization in both MT24 and MT24M fibroblasts compared to controls, as quantified by colocalization analysis (Manders’ coefficient M1; *p* < 0.05; Figure 5B). This increased mitochondria–lysosome association is compatible with enhanced mitophagy.

To corroborate these findings, we quantified mitochondrial DNA copy number by quantitative PCR, normalizing mt-ND1 and mt-ND5 mtDNA levels to RPPH1 nuclear DNA. Consistent with a reduced mitochondrial mass, mtDNA copy number was significantly reduced in both mutant fibroblast lines, with a decrease of approximately 25% in MT24 and 35% in MT24M cells relative to controls (Figure 5C).

Collectively, these data indicate that the m.1014A>G variant is associated with increased mitochondria–lysosome association, possibly reflecting enhanced mitophagic turnover of dysfunctional mitochondria, and with a reduction in mitochondrial mass, which may contribute to the observed respiratory defects.

## 3. Discussion

This study aimed to functionally characterize the m.1014A>G variant in the *MT-RNR1* gene (encoding for 12S rRNA), identified as homoplasmic in blood and fibroblasts of a case index and her mother. The proband presents with severe early-onset drug-resistant focal epilepsy who remained without a definitive molecular diagnosis for more than three decades. Re-evaluation through dedicated mitochondrial DNA analysis identified a novel homoplasmic *MT-RNR1* variant, m.1014A>G, which was also detected in the mother with milder phenotypes. Functional studies in patient-derived fibroblasts—including mitochondrial rRNA quantification, quantitative mitoproteomics, high-resolution respirometry, and mitochondrial network analysis—consistently pointed to a defect of mitoribosome biogenesis and function. Notably, whereas *MT-RNR1* variants have in some cases been reported as co-occurring alongside other candidate pathogenic variants or disease risk modifiers without a clearly established independent causal role (e.g., as a risk factor for diabetes-associated hearing loss, in co-segregation with MT-ATP6 variants underlying cardiac pathology, or as an accessory finding in MERRF syndrome) [30,31,32], no other pathogenic variant was identified in this family despite extensive nuclear genetic testing, and the convergent structural and functional abnormalities reported here support a likely contribution of m.1014A>G to the patient’s phenotype, although a direct cause–effect relationship remains to be formally established. Together, these findings expand the phenotypic spectrum of *MT-RNR1*-associated disease beyond canonical aminoglycoside-related hearing loss and highlight the importance of dedicated mtDNA analysis in unexplained epileptic encephalopathies.

The mitochondrial 12S and 16S rRNAs are co-transcribed from a single polycistronic transcript and mature through a coordinated pathway of processing, modification, and progressive incorporation into the assembling small ribosomal subunit (mt-SSU) [33,34]. The m.1014A>G substitution localizes to helix H23, a central-domain element of the mt-SSU platform that undergoes active remodeling during assembly, distinct from h44/h45, which form the decoding center and mediate aminoglycoside binding. Variants affecting h23 are therefore expected to impair ribosome biogenesis rather than decoding-site function.

Consistent with this, static structural modeling showed that m.1014A>G creates a novel close contact with the platform protein bS21m (MRPS21), compacting the nucleotide–protein interface around position 1014. Because h23 docking and incorporation of the bS21m–uS11m module are coupled steps in mt-SSU platform maturation [25,35], this predicts a defect in mt-SSU assembly rather than in decoding function. Three independent lines of evidence support this prediction: the altered 12S:16S rRNA ratio, consistent with accumulation of an unassembled 12S transcript; the absence of gentamicin-induced hypersensitivity; and the pronounced changes in the mitochondrial proteome. Indeed, quantitative mitoproteomics provided the most comprehensive view of the consequences of m.1014A>G, showing that nearly all detected mt-SSU proteins were significantly reduced in abundance, whereas mt-LSU proteins were affected to a lesser extent, similar to what has been previously reported for pathogenic defects in mt-SSU biogenesis factors [36].

Defective mt-SSU assembly in turn impairs mitochondrial translation, as supported by reduced abundance of mtDNA-encoded respiratory-chain subunits. The reduction in nuclear-encoded mitochondrial proteins may reflect increased mitochondrial turnover and the associated reduction in mitochondrial mass, as discussed below.

Mitoribosomal dysfunction results in OXPHOS deficiency and bioenergetic failure [14,37]. High-resolution respirometry demonstrated that this molecular defect translates into a significant bioenergetic phenotype. While routine respiration was relatively preserved, maximal electron transport system capacity was markedly reduced, indicating loss of mitochondrial reserve capacity. This distinction is particularly relevant in neurons, which heavily rely on oxidative phosphorylation to meet sudden increases in energy demand. During epileptic activity, ATP requirements rise dramatically; cells with limited respiratory reserve may therefore be unable to sustain ion homeostasis and synaptic function, creating a permissive environment for neuronal hyperexcitability [38,39,40]. We therefore propose, as a plausible mechanistic interpretation, that impaired mitoribosome function and the resulting loss of respiratory reserve may contribute to the severe epileptic phenotype observed in the proband; this hypothesis remains to be tested in neuronal models. This mechanism parallels that of nuclear-encoded mt-SSU assembly defects (e.g., MRPS16, MRPS22, MRPS25, MRPS34), which similarly cause combined OXPHOS deficiency and encephalopathies frequently featuring epilepsy [41,42,43,44]. However, to our knowledge, this is the first report of an epileptic phenotype associated with a candidate pathogenic variant in the 12S rRNA itself, rather than in a nuclear-encoded mitoribosomal protein.

Mitochondrial network analysis further demonstrated marked fragmentation of the mitochondrial reticulum. This pattern is characteristic of mitochondrial stress and is commonly observed in the setting of impaired OXPHOS function and partial membrane depolarization [45]. Together with the overall reduction in mitochondrial protein abundance and mtDNA copy number, these findings are compatible with activation of compensatory quality-control pathways, including enhanced mitophagy, as suggested by the increased mitochondria–lysosome colocalization, in response to accumulation of dysfunctional mitochondria.

Taken together, our data point to a previously unrecognized candidate disease mechanism within *MT-RNR1* that affects mitochondrial translation and respiratory reserve capacity and may contribute to the patient’s severe epilepsy [16], possibly related to neuronal energy deficit and progressive neurodegeneration that may occur in mitochondrial diseases.

Nevertheless, several clinical aspects remain unresolved. Despite homoplasmy in peripheral blood and fibroblasts, the proband and her mother exhibited markedly different clinical phenotypes. Similar variability is well documented for other *MT-RNR1* disorders and likely reflects the influence of nuclear modifier genes, tissue-specific compensatory mechanisms, and differences in organ vulnerability [15]. This case also highlights an important limitation of routine exome-based diagnostics [6]. Despite previous extensive testing, the diagnosis emerged only after dedicated mtDNA analysis more than three decades after disease onset. The prolonged attribution of the phenotype to tuberous sclerosis complex, despite a lack of clinical consistency, further illustrates the risk of premature diagnostic closure. Our findings support inclusion of mtDNA analysis in WES-negative epileptic encephalopathies, particularly when maternal inheritance or multisystem involvement is present.

The different clinical expression of m.1014A>G in the proband and her mother deserves specific consideration. The mother’s clinical history (a cavernous angioma, a cerebellar hemorrhagic stroke with slowly progressive ataxia, anxiety/depression, sleep disturbances and chronic macrocytosis) is milder than, and qualitatively different from, the proband’s epileptic encephalopathy, but remains compatible with a mitochondrial disorder. Variable expressivity and incomplete penetrance are well-recognized features of homoplasmic mtDNA variants, including *MT-RNR1* variants, in which carriers within the same maternal lineage may range from asymptomatic to severely affected. Several non-mutually exclusive factors may account for this variability: (i) nuclear genetic modifiers, including variants in genes involved in mitoribosome assembly, rRNA modification or mitochondrial quality control; (ii) tissue-specific differences in mtDNA copy number, rRNA processing and compensatory responses, as homoplasmy in blood and fibroblasts does not exclude tissue-specific differences in the downstream biochemical consequences of the variant; and (iii) additional environmental or acquired factors, such as the perinatal events and the cortical dysplasia documented in the proband, which may have acted as a ‘second hit’. In this context, the focal nature of the epilepsy suggests that the mitochondrial defect may have lowered the threshold for seizure generation in the presence of these local structural abnormalities. Notably, the biochemical defect in fibroblasts was qualitatively similar in both carriers and only partially milder in the mother, indicating that the severity of the cellular phenotype does not fully parallel the clinical phenotype and supporting the contribution of tissue-specific and/or individual modifying factors. Neuron-specific susceptibility could be directly addressed in patient-derived induced pluripotent stem cell (iPSC)-derived neurons or brain organoids, which is, however, beyond the scope of the present work.

Analysis of the pedigree is suggestive of maternal inheritance (Figure 1A). In addition to the mother, whose phenotype is milder but compatible with mitochondrial disease, the maternal aunt (III-3) died at 8 years of age of unknown causes, the maternal grandmother (II-2) developed late-onset hearing loss, and a maternal great-aunt (II-3) was affected by diabetes. Although mild or non-specific, these features are all recognized manifestations of mitochondrial disorders, and late-onset hearing loss in particular is the hallmark of *MT-RNR1* variants; together, they fit the variable expressivity typical of these conditions. Unfortunately, no DNA samples from other maternal relatives were available for segregation analysis; therefore, maternal transmission of m.1014A>G cannot be formally documented, and a de novo origin in the mother’s germline cannot be excluded. Analysis of further maternal relatives, should they become available, will be important to clarify penetrance and the clinical spectrum associated with this variant.

The combination of population data, structural considerations, and convergent functional abnormalities is suggestive of a pathogenic role of m.1014A>G. According to current mtDNA-specific ACMG/AMP recommendations [46], the variant meets PM2_Supporting (absent from gnomAD and HelixMTdb and extremely rare in MITOMAP) and the functional criterion PS3, based on studies in patient-derived fibroblasts demonstrating a damaging effect, although a contribution of the nuclear genetic background to the observed defect cannot be formally excluded. Because this functional evidence derives exclusively from patient-derived fibroblasts and has not been confirmed in transmitochondrial cybrids or by single-fiber analysis, PS3 was applied at the supporting level only (PS3_Supporting). No additional criteria could be applied: PP1 is not applicable, because segregation could not be assessed in other maternal relatives, and PP3 could not be used, because no MitoTIP, HmtVar or APOGEE2 predictions are available. On this basis (PM2_Supporting + PS3_Supporting), m.1014A>G is currently classified as a variant of uncertain significance (VUS); cybrid studies, which would allow PS3 to be applied at a higher strength, and the identification of additional unrelated carriers with a compatible phenotype will be required before the variant can be classified as likely pathogenic. If confirmed, recognition of m.1014A>G as an *MT-RNR1*-related mitochondrial disorder also has immediate clinical relevance. Although the variant does not appear to confer the aminoglycoside toxicity characteristic of canonical *MT-RNR1* decoding-center variants, avoidance of potentially mitochondriotoxic medications (including antiepileptic drugs such as valproic acid) remains advisable, and mitochondrial disease-specific management strategies should be considered [7,8,16].

We acknowledge several limitations of this study. First, the findings are based on a single family with only two carriers, and segregation analysis in additional maternal relatives was not possible. Second, the clinical manifestations were heterogeneous between mother and daughter, which limits genotype–phenotype inferences. Third, functional studies were performed in dermal fibroblasts, which may not recapitulate the neuron-specific consequences of the variant; iPSC-derived neuronal models will be required to address this aspect in future studies. Fourth, and most importantly, in the absence of transmitochondrial cybrid studies, the biochemical defect cannot be attributed to m.1014A>G alone, as a contribution of the nuclear genetic background or of other mtDNA variants of the haplogroup cannot be excluded.

In conclusion, we report the first association between the homoplasmic *MT-RNR1* variant m.1014A>G and severe epileptic encephalopathy. Functional studies in patient-derived fibroblasts suggest that the variant impairs mt-SSU assembly, resulting in impaired mitochondrial translation, respiratory-chain dysfunction, and reduced bioenergetic reserve capacity. These findings expand the phenotypic spectrum of *MT-RNR1* disease beyond aminoglycoside-associated hearing loss and point to mitoribosome assembly failure as a candidate novel disease mechanism within *MT-RNR1*. More broadly, this case emphasizes the importance of dedicated mtDNA analysis in unexplained epilepsy, even after extensive inconclusive nuclear genetic testing.

## 4. Materials and Methods

### 4.1. Sequencing and Bioinformatic Analysis

Genomic DNA from peripheral blood of the index case and both her parents was enriched for the coding regions and exon–intron boundaries of approximately 22,000 nuclear genes plus all of the mitochondrial genome using the 4bases WholEx Pro v3 capture kit (4bases SA, Manno, Switzerland) and sequenced on the Illumina NextSeq 550Dx platform (paired-end, 2 × 150 bp) (Illumina, San Diego, CA, USA), according to manufacturer protocols.

Sequence alignment, variant calling, annotation, and copy number variant (CNV) analysis were performed using VarSome Clinical v11.9 (Saphetor SA, Lausanne, Switzerland), with GRCh38/hg38 as the reference genome. Variant interpretation and classification were conducted according to the recommendations of the American College of Medical Genetics and Genomics and the Association for Molecular Pathology guidelines [18].

Tertiary analysis of the sequencing data was conducted using two independent strategies: a panel-based study (an internally assembled diagnostic set of 1688 genes reported to be associated with monogenic neurodevelopmental disorders and epilepsies was used, with 1645 genes meeting a quality threshold of ≥95% of target bases covered at a sequencing depth of ≥20×) and a gene-agnostic trio study, with additional manual review of de novo variants.

### 4.2. Quantification of Heteroplasmy

To quantify the heteroplasmy level of the m.1014A>G variant in fibroblasts of the proband (MT24) and her mother (MT24M), DNA was extracted using the Puregene Blood Kit (Qiagen, Hilden, Germany, 158023) according to the manufacturer’s instructions. A fragment spanning the variant site was amplified by PCR using the Phusion High-Fidelity PCR Master Mix (Thermo Fisher Scientific, Waltham, MA, USA, F531S) with the following primers: *MT-RNR1* forward 5′-GGG TTG GTC AAT TTC GTG CC-3′ and *MT-RNR1* reverse 5′-CGG ACG GGG CTG GGG CTG ATG TGT GTG-3′. The PCR product was subsequently subjected to Sanger sequencing to assess the proportion of mutant versus wild-type alleles.

### 4.3. Assessment of mtDNA Copy Number

Mitochondrial DNA copy number (mtDNA-CN) was quantified by semi-quantitative TaqMan real-time PCR using the TaqPath ProAmp Master Mix (A30866, Thermo Fisher Scientific) in a final reaction volume of 10 μL on the CFX Opus Real-Time PCR System (Bio-Rad, Hercules, CA, USA). Two mitochondrially encoded targets were used: *MT-ND1* (Hs02596873_s1, VIC) and *MT-ND5* (Hs02596878_g1, VIC). The nuclear non-coding RNA gene *RPPH1* (Hs03297761_s1, FAM), present at two copies per diploid genome, was used as the internal reference. Relative mtDNA copy number was calculated as the ratio of mitochondrial to nuclear target signals and expressed relative to control cells.

### 4.4. Cell Culture

Primary skin fibroblasts were isolated from skin biopsies performed on the two patients (mother and daughter) by mechanic and enzymatic digestion, respectively, using a scalpel and enzyme digestion like collagenase V (#C9263, Sigma-Aldrich, St. Louis, MO, USA) and TrypLE (Thermo Fisher Scientific). Control fibroblasts were derived from two healthy male donors, obtained as surgical waste material: CTRL1 from foreskin tissue collected during circumcision (4 years of age) and CTRL2 from skin collected during scoliosis surgery (10 years of age). All control and patient fibroblast lines were used between passages 4 and 10. After that, fibroblasts were cultured and expanded in vitro in high-glucose Dulbecco’s Modified Eagle’s Medium (DMEM, Euroclone, Milan, Italy) supplemented with 10% Fetal Bovine Serum (FBS, Euroclone) and 1% penicillin/streptomycin 100X (PenStrep, Euroclone). Primary HDF cultures were maintained at 37 °C and 5% CO_2_, and they were monthly tested with a PCR to verify that they were mycoplasma-free.

### 4.5. RNA Extraction and Quantitative Real-Time PCR

A total of 250,000 cells were seeded on 50 mm plates and harvested after 24 h in TriFast reagent (Euroclone) for total RNA extraction following the manufacturer’s instructions. RNA concentration, quality, and purity were assessed using a NanoDrop ND-1000 spectrophotometer (NanoDrop Technologies, Wilmington, DE, USA). One microgram of total RNA was reverse-transcribed using the iScript Advanced cDNA Synthesis Kit (Bio-Rad, Hercules, CA, USA, #1725037). Quantitative PCR was performed using iTaq Universal SYBR Green Supermix (Bio-Rad, #1725120) on the CFX Opus Real-Time PCR System (Bio-Rad). Each experiment was performed at least three times, with each sample representing the mean of a technical duplicate. Relative gene expression was calculated using the 2^−ΔΔCt^ method, with H3 as the normalization reference. The following primer sequences were used:
**Target****Direction****Sequence (5′→3′)**MT-RNR1 (12S)ForwardTAGAGGAGCCTGTTCTGTAATCGATMT-RNR1 (12S)ReverseCGACCCTTAAGTTTCATAAGGGCTAMT-RNR2 (16S)ForwardGCCTGCCCAGTGACACATGMT-RNR2 (16S)ReverseCACGGGCAGGTCAATTTCACMT-RNR1 1014ForwardGGG TTG GTC AAT TTC GTG CCMT-RNR1 1014ReverseAGGGCTAAGCATAGTGGGGTH3ForwardGAAGAAACCTCATCGTTACAGGCCTGGTH3ReverseCTGCAAAGCACCAATAGCTGCACTCTGGAA

### 4.6. Gentamicin Cytotoxicity Assay

The in vitro cytotoxicity of gentamicin (aminoglycoside antibiotic, #15750037, Thermo Fisher Scientific) on primary HDFs from patients and healthy controls was tested through Crystal Violet staining, used for screening cell viability under different concentrations of the antibiotic (1 and 5 mM) at different endpoints (24, 48 and 72 h). The antibiotic was added at the right concentration to the culture medium Dulbecco’s Modified Eagle’s Medium (DMEM, Euroclone) supplemented with 10% Fetal Bovine Serum (FBS, Euroclone) and 1% penicillin/streptomycin 100X (PenStrep, Euroclone). After 24, 48 and 72 h, HDFs were stained with 10% Crystal Violet in Phosphate-Buffered Saline 1X (PBS, Euroclone), incubated for 30 min at 37 °C, washed twice in Milli-Q water and then lysed with 1% SDS in PBS. The absorbance of Crystal Violet (corresponding to viable cells) was measured at 600 nm using the Multiskan SkyHigh Microplate Spectrophotometer (Thermo Fisher Scientific).

### 4.7. Mitochondrial Proteome Analysis

#### 4.7.1. Mitochondrial Isolation for Mitoproteome

Mitochondria from patients and control HDFs were isolated using the Mitochondria Isolation Kit for cultured cells (#89874, Thermo Fisher Scientific) according to the manufacturer’s instructions. Briefly, ~3.5 × 10^6^ cells were centrifuged at 200× *g* for 5 min, lysed and subjected to differential centrifugation at 700× *g* for 10 min and 12,000× *g* for 15 min at 4 °C. The mitochondrial pellet was washed once and boiled in Pierce^TM^ RIPA buffer (89900, Thermo Fisher Scientific) at 95 °C for 5 min. Finally, the Pierce™ BCA Protein Assay Kit (23250, Thermo Fisher Scientific) was used for protein quantification before digestion.

#### 4.7.2. Mitoproteome Sample Preparation and Data Acquisition

A total of 11 µg of protein per sample was subjected to enzymatic digestion using the EasyPep™ MS Sample Prep Kit (Thermo Fisher Scientific, Waltham, MA, USA), following the manufacturer’s instructions. The resulting peptide mixtures were analyzed by nano-ultra-high-performance liquid chromatography coupled to high-resolution mass spectrometry (nUHPLC-HRMS). Chromatographic separation was performed on an Ultimate 3000 nanoLC system (Thermo Fisher Scientific, Bremen, Germany) interfaced with an Orbitrap Fusion Lumos Tribrid mass spectrometer (Thermo Fisher Scientific) equipped with an ESI source. Peptides were first loaded onto a PepMap trap column (1 µL injection volume, flow rate of 60 µL/min, 1.0 min loading time) and subsequently separated on a C18 reversed-phase analytical column (EasySpray PepMap, Thermo Fisher Scientific; 250 mm × 75 µm inner diameter, 2.0 µm particle size, 100 Å pore size and column flow of 0.3 µL/min) using a linear 60 min gradient. Data were acquired in data-independent acquisition (DIA) mode. Full MS scans were collected at a resolution of 60,000, followed by DIA scans acquired in the Orbitrap at a resolution of 30,000 using 10 Da isolation windows. The automatic gain control (AGC) target and maximum ion injection time were set to custom values of 200 ms and 40 ms, respectively, with a loop count of 30. Peptide fragmentation was achieved by higher-energy collisional dissociation (HCD) with a normalized collision energy of 30%. The AGC target was set to 250% in MS1 with the maximum injection time set to auto, and the event DIA AGC target was set to 800% with the maximum injection time set to auto.

#### 4.7.3. Data Processing and Protein Identification

DIA raw data were processed using Spectronaut version 20 (Biognosys AG, Schlieren, Switzerland), applying the DirectDIA (deep learning) workflow for peptide and protein identification. Carbamidomethylation of cysteine was set as a fixed modification, while N-terminal protein acetylation and methionine oxidation were included as variable modifications. A false discovery rate (FDR) threshold of <1% was applied at both the peptide and protein levels, and proteins were considered confidently identified when supported by at least one unique peptide. Relative label-free quantification was performed based on peptide ion intensities matched against a spectral library generated in silico. Data normalization across runs was carried out using Spectronaut’s built-in global normalization algorithm. Retention time alignment was achieved through a non-linear regression model based on indexed retention time (iRT) peptides, ensuring high-precision peptide identification and quantification across all LC-MS runs. To ensure the reliability of downstream quantitative analyses, proteins presenting missing intensity values across samples were excluded from further consideration.

### 4.8. Western Blot Analysis

Approximately 250,000 fibroblast cells were seeded in a 60 mm plate for 24 h and lysed in the Pierce^TM^ RIPA buffer (#89900, Thermo Fisher Scientific) with the addition of Halt^TM^ Protease Inhibitor Cocktail 100X (#87786, Thermo Fisher Scientific). Lysates were quantified through the Bradford assay (#500-0006, Bio-Rad) and the same quantity of proteins (~20 µg) was resolved by SDS-PAGE using precast gels (NW04122BOX, Thermo Fisher Scientific). The separated proteins were transferred from the gel to a nitrocellulose membrane (#1704158, Bio-Rad) and incubated with the following primary antibodies: MT-CYB polyclonal antibody (55090-1-AP, Proteintech, Rosemont, IL, USA), TOM20 polyclonal antibody (11802-1-AP, Proteintech), VDAC polyclonal Antibody (PA1-954A, Thermo Fisher Scientific), MTCO1 polyclonal antibody (PA5-115081, Thermo Fisher Scientific), MT-ND1 polyclonal antibody (PA5-120599, Thermo Fisher Scientific) and HSP90 rabbit monoclonal antibody (4877S, Cell Signaling Technology, Danvers, MA, USA) used as a normalizer. The secondary antibody used was the HRP-linked anti-rabbit IgG antibody (7074S, Cell Signaling Technology). To allow protein detection, the reagent Clarity Max Western ECL Substrate (1705062, Bio-Rad) was used, the chemiluminescent images were acquired with the ChemiDoc MP Imaging System (Bio-Rad), and the analysis was carried out using the software ImageJ (Fiji, version 2.16.0/1.54t) to quantify the intensity of protein bands [47].

### 4.9. Cell Imaging

MitoRed staining: A total of ~50,000 HDFs were seeded on glass coverslips. Almost 24 h later, they were washed twice in PBS (Euroclone) and subsequently stained by adding the fluorescent rhodamine-based dye Mito Red (#53271, Merck, Darmstadt, Germany), diluted at the final concentration of 200 nM in Hank’s Balanced Salt Solution (HBSS, Euroclone). The incubation with the dye was performed for 30 min at 37 °C and, successively, cells were washed twice in PBS and fixed with paraformaldehyde 4% (#J61899.AK, Thermo Fisher Scientific) for 15 min. Nuclei were counterstained with DAPI (#10236276001, Roche, Mannheim, Germany) and the coverslip was mounted on glass slides. The slides were visualized using the Zeiss LSM900 confocal microscope by ZEN software v3.8 (blue edition) (Zeiss, Oberkochen, Germany), analyzing ~50 cells for each sample type. Finally, analysis of confocal images was carried out with the ImageJ mitochondria analyzer v2.3.1 (Fiji) [47,48].

MitoTracker Green and LysoTracker Red staining: A total of ~5000 HDFs per well were seeded in a 96-multiwell plate. 24 h later, they were washed twice in PBS and stained with the fluorescent dye MitoTracker™ Green (#M7514, Thermo Fisher Scientific) at a final concentration of 100 nM and with the probe LysoTracker™ Red DND-99 (#L7528, Thermo Fisher Scientific) at a final concentration of 50 nM in HBSS. The incubation was performed for 30 min at 37 °C and then cells were washed twice in PBS. Image acquisition was carried out with the Cytation 5 cell imaging system paired with a BioSpa 8 automated incubator, using Gen5 software version 3.10 (Agilent Technologies, Santa Clara, CA, USA). Finally, colocalization analysis was performed with the JACoP plugin v2.0 for ImageJ (Fiji) [47,49], and mitochondria–lysosome colocalization was quantified as Manders’ coefficient M1 (fraction of LysoTracker signal overlapping MitoTracker Green signal), analyzing ~50 cells for each sample type.

### 4.10. High-Resolution Respirometry

High-resolution respirometry (HRR) was performed using the instrument Oroboros NextGen-O2k (Oroboros Instruments, Innsbruck, Austria), in order to measure the oxygen consumption of HDFs from the two patients and healthy control.

A total of ~2 million HDFs (from patients and control) were collected, centrifuged (200× *g* for 5 min) and resuspended in the mitochondrial respiration medium MiR05 (0.5 mM EGTA, 3 mM MgCl2, 60 mM lactobionic acid, 20 mM taurine, 10 mM KH_2_PO_4_, 20 mM HEPES, 110 mM d-sucrose, 1 g/L BSA fatty acid-free; Oroboros Instruments), prepared using the MiR05-Kit (Oroboros Instruments).

The concentration of O_2_ was measured inside the two chambers of the instrument, following the protocol substrate-uncoupler-inhibitor titration (SUIT) “001 O_2_ ce-pce D003”. Firstly, the routine respiration of HDFs was recorded (only relying on the presence of their own endogenous substrates). After that, HDFs were permeabilized with 1 µg of digitonin and sequentially treated with a series of substrates, in order: pyruvate and malate (leak respiration), ADP (OXPHOS capacity), cytochrome c (mitochondrial membrane integrity), uncoupler CCCP and glutamate (electron transfer capacity), succinate and octanoylcarnitine (electron flow through Complexes I and II), rotenone (electron transfer capacity downstream of Complex I, since inhibited), glycerophosphate (electron transfer capacity via Complexes III and IV), antimycin A (residual oxygen consumption), ascorbate and TMPD (CIV activity), and lastly the sodium azide (correction for chemical background). Finally, the O_2_ consumption was assessed using the software DatLab 8 (Oroboros Instruments), which permits computing the flux of O_2_ by representing it as the negative derivative of the O_2_ concentration in real-time.

### 4.11. Statistical Analyses and Reproducibility

All the experiments are representative of at least three independent replicates. Statistical tests were performed and heatmaps were generated using GraphPad Prism 10.2.0 (GraphPad Software, Boston, MA, USA), as indicated in figure legends. The reported blot and micrographs are representative of three independent experiments.

## Data Availability

The original contributions presented in this study are included in the article/Supplementary Materials. Further inquiries can be directed to the corresponding authors.

## Supplementary Materials

The following supporting information can be downloaded at https://www.mdpi.com/article/10.3390/ijms27219513/s1.
