# Supplementary material for: Impaired Mitochondrial Translation and Bioenergetic Dysfunction Associated with the MT-RNR1 m.1014A>G Variant
Source: Int J Mol Sci. 2026 Oct 24;27(21):9513. doi: 10.3390/ijms27219513 (PMC13762810; doi:10.3390/ijms27219513)
Supplement: Supplementary file 1 [file ijms-27-09513-s001.zip › Supplementary Figures.pdf]

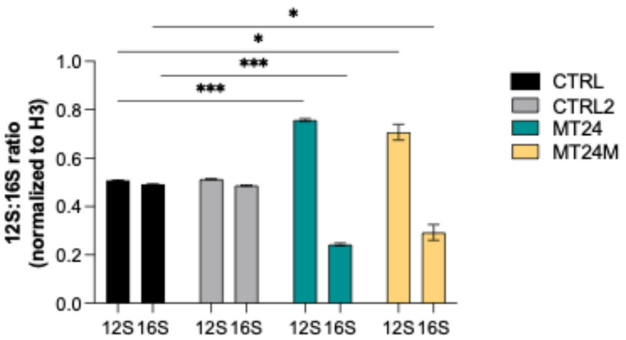

Supplementary Figure S1. Quantification of 12S and 16S mt-rRNA levels using primers spanning the m.1014A>G mutation site.

Expression levels of 12S and 16S mt-rRNA determined by quantitative RT-PCR site in primary HDFs from CTRL, CTRL2, MT24, and MT24M using primer pair designed to include the m.1014A>G mutation and normalized to histone H3. The 12S:16S ratio is displayed for each cell line. Data represent mean ± SEM. Statistical significance: ***$p < 0.001$; *$p < 0.05$ (2way ANOVA)

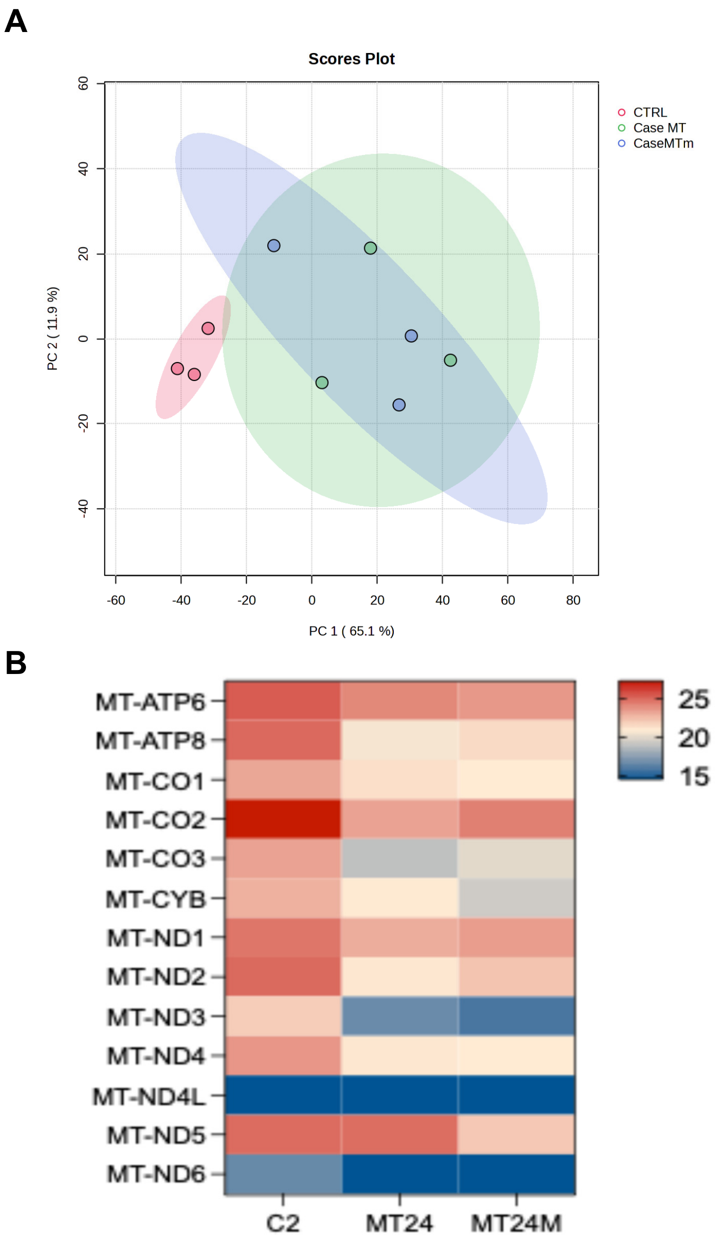

Supplementary Figure S2. Proteomic quality control and mtDNA-encoded protein expression.
(A) Principal component analysis (PCA, left panel; PC1 65.1%, PC2 11.9%) and partial least squares discriminant analysis (PLS-DA, right panel; Component 1 25.9%, Component 2 12.7%) of the full proteomic dataset for CTRL (red), MT24 (green, Case MT), and MT24M (blue, CaseMTm). Ellipses represent 95% confidence intervals. The clear separation between groups confirms a distinct proteomic signature in both affected individuals relative to controls, with MT24M showing an intermediate profile.
(B) Heatmap of Log_2 LFQ intensities of the 13 mtDNA-encoded OXPHOS subunits (MT-ATP6, MT-ATP8, MT-CO1–3, MT-CYB, MT-ND1–6, MT-ND4L) across CTRL2 (C2), MT24, and MT24M. Color scale ranges from blue (low) to red (high).
